# Supplementary material for: Exploring the challenges in the management of childhood pneumonia-qualitative findings from health care providers from two high prevalence states in India
Source: PLOS Glob Public Health. 2022 Aug 22;2(8):e0000632. doi: 10.1371/journal.pgph.0000632 (PMC10021893; doi:10.1371/journal.pgph.0000632)
Supplement: S1 Text — (DOC) [file pgph.0000632.s001.doc]

**Care seeking in Childhood Pneumonia Management: An Exploratory Study**

**Semi Structured Interview Guide- Health Care Providers- (Govt. Doctor and Community Health Workers)**

1. Demographic details: (Age, gender, length of service in current position)
2. What are your perceptions about the prevalence of pneumonia in children under 5, more specifically can you tell me about childhood pneumonia in this area*? (Probe: whether he/she thinks it is a big problem in this area, on what basis is this said)*
3. How much do you think the people in this community are aware about pneumonia in children under 5? (*Probe: whether MO/CHW is really aware about this, on what basis does he/she say this)*
4. What do you think people in this area usually do for seeking care for their children with pneumonia and why do they usually do this? (*Probe: all types of care sought, Allopathic-govt. or pvt. Traditional, Indian medicine-which type, home remedies etc)*
5. How do you go about diagnosing pneumonia in children under 5? *(Probe: sequence of steps taken, any investigations ordered and why)*
6. What kinds of challenges (if any) do you face in diagnosing pneumonia in children under 5? *(Probe: whether these are due to inadequate facilities in his/her facility, not adequately trained staff, poor awareness in community about pneumonia, delay in care seeking etc)*
7. What are your beliefs about delays in recognition of illness by families, or delays in seeking care due to affordability, accessibility by families or even any delays in getting treatment at your health facility?
8. Please describe how you usually go about treating children under 5 diagnosed with pneumonia? *(Probe: sequence of steps once diagnosis made)*
9. How satisfied are you with the care provided in your facility for the management of pneumonia in children under 5?
10. What do you believe are some challenges/barriers you face in the treatment/management of children under 5 with pneumonia in your area? *(Probe: inadequate drugs, poor infrastructure, not enough trained staff, poor compliance with treatment protocols by families, poor follow-up by families etc)*
11. What are your perceptions on promotive, preventive, curative approaches in the management of pneumonia? *(Probe: how he/she classifies promotive, preventive and curative approaches, what is his/her understanding of each of these and their pros and cons)*
12. Probe about any CME programmes attended, trainings received, referral system used, adequacy of medicines/equipments
13. Do you have any suggestions/ recommendations for improving care for under 5 children with pneumonia

*Thank You*
