# Supplementary material for: Exploring the challenges in the management of childhood pneumonia-qualitative findings from health care providers from two high prevalence states in India
Source: PLOS Glob Public Health. 2022 Aug 22;2(8):e0000632. doi: 10.1371/journal.pgph.0000632 (PMC10021893; doi:10.1371/journal.pgph.0000632)
Supplement: S2 Text — (DOC) [file pgph.0000632.s002.doc]

**Code Book : HCP Perceptions (Nvivo Vervsion 8)**

**HCP-Community awareness of pneumonia**

HCP-Community-Pneumonia deaths in community

HCP-Community-what families do in case of Pneumonia

HCP Community- Perceptions about community awareness of Pneumonia

HCP- Community- How big a problem

HCP-Community -Perceptions on common health problems in community

**HCP-Diagnosis of pneumonia**

HCP- Diag.- Steps in pneumonia diagnosis

HCP-Diag- HCP awareness about symptoms of Pneumonia

HCP-Diag- Practice followed in treating pneumonia

HCP-Diag.- Challenges faced in diagnosis of pneumonia

HCP-Diag-Expectations and suggestions

HCP-Diag-First treatment or advise given for mother with sick children

HCP-Diag-Referrals to higher centres

**HCP-Perceptions on care seeking for pneumonia in community**

HCP- Care- For what kinds of illnesses do they go to UCPs

HCP- Care- Public- Private care seeking

HCP- Care-Home deliveries vs instituitional deliveries

HCP- Care-Where treatment sought and why

HCP-Care- Decision making in care seeking

HCP-Care- Delays in recognition of illness, delays in care seeking by community

HCP-Care- Issues of follow-up, adherence

HCP-Care- Perceptions on roles of parents-families in recognizing illness

HCP-Care- What people in community do to manage pneumonia, other illnesses

HCP-Care-Perceptions on gender differences in care seeking

HCP-Care-Perceptions on public attitudes towards HCPs

**HCP-Perceptions on preventive, promotive, curative approaches in pneumonia care**

HCP-Approaches- Breast feeding, immunization, nutrition-information given

HCP-Approaches- HCP classification of these 3 approaches and understanding of it

HCP-Approaches-Exclusive breast feeding practice in community

**HCP- Challenges faced in care delivery in community and in facility**

HCP-Challenges-Alternative medicines used

HCP-Challenges-Community use of home remedies

HCP –Challenges- Role of UCPs in care provision

HCP-Challenges- Community trust in UCPs

HCP-Challenges- Quality of facilities and services available in govt facilities

HCP-Challenges- Issues concerning availability of medicines, other equipments

HCP-Challenges- Training and supervision in pneumonia management-what is done

HCP- Challenges - Need for more training, why?

HCP-Challenges- IMNCI training received, or any other training in child care

HCP-Suggestions for improving care
