# Supplementary material for: Exploring the challenges in the management of childhood pneumonia-qualitative findings from health care providers from two high prevalence states in India
Source: PLOS Glob Public Health. 2022 Aug 22;2(8):e0000632. doi: 10.1371/journal.pgph.0000632 (PMC10021893; doi:10.1371/journal.pgph.0000632)
Supplement: Attachment — Submitted filename: Response to Reviewers .docx [file pgph.0000632.s003.docx]

**Response to Comments of Reviewers**

**Additional Editor Comments**

Thank you for submitting this important manuscript to us. All three reviewers agree that this is a critical study and a well-written manuscript that would interest PLOS GPH readers. They provide valuable recommendations and suggestions to help improve the manuscript to make it suitable for publication. It is imperative to attend to reviewers’ comments. Some significant revisions include, but are not limited, to the following:

**Comment 1.** In the introduction and perhaps in the discussion, provide more context about the health system and its management structures for readers who may not be as familiar with the situation in the study area. This also would help make study findings and recommendations better tailored for implementation.

Response: Thank you for this suggestion. We have now included a brief write-up about the rural health system in India (Pages 5-6; Lines 119-146)

**Comment 2**. In the methods section, clarify the relationship between the larger study and the generation of this particular manuscript. Further, it would be useful to connect the theory or theories driving the study questions (e.g., health systems, community-based engagement) and how it/they connect(s) to the methods used. Use these theories to then in the interpretation of findings in the discussion. It might also be helpful to include some sample interview questions and codes, even as supplementary materials or as a table. Uploading the entire codebook is not necessary.

Response: We do appreciate these suggestions and in pursuance of this have included text towards the end of the introduction section explaining the larger study of which this current one on the perceptions of health care providers was one component (Page 4; Lines 87-99). We have also introduced a section titled ‘Conceptual Framework’ wherein we have provided a description of the Andersen and Newman framework for health service utilization (2005). This guided our study and was used to explain our study findings (Pages 4- 5; Lines 105-117). In the methods section we have provided brief text which explains how the sites were selected and its links to the larger study (Page 6; Lines 148-155). As suggested we have included a copy of the guide we used as a supplementary file and have elaborated a little more on the nature of questions asked under the section on ‘Study Instruments’ (Page 7; Lines 163-170)

**Comment 3.** One reviewer stated that the results section as currently written is difficult to follow. You could revise to better reflect broader categories and sub-themes and integrate the quotes accordingly. The text should read more narratively, rather than a presentation of block quotes divorced from the summary. Additionally, it might be helpful to compare and contrast perspectives from different participant groups to understand the similarities and specific differences between them.

Response: Thank you for these suggestions which have been most helpful. We have now reorganized our results using the Andersen- Newman framework, deleted findings that were repetitive, have re-named some of our thematic heads and incorporated quotes within the narratives all of which we believe have made our results easier to understand.

**Comment 4**. In the discussion section, reviewers have provided some helpful comments on how to structure this section such as beginning with a summary paragraph and then with a discussion of each of the key points in the following paragraphs. The manuscript can also be strengthened by linking study outcomes to specific interventions that have been successful in similar communities. Finally, it might be helpful to explore additional questions that remain unclear where further research is needed.

Response: Thank you, we agree and have revised our discussion section based on these comments. We begin first with the key findings identified followed by a discussion of these findings and have introduced additional text comparing with other studies

:

**Reviewers' comments:**

**Reviewer #1:** The paper is very well written. There is so much rich content that might inform the improvement of management of pneumonia in similar settings.
Just a few comments:

**Comment 1.** The authors need to provide further detail about the organization of the health system. This is good for further contextualizing this phenomena and facilitates Knowledge translation. For instance, who is mandated to oversee healthcare provision in the states? Is it a decentralized system?

Response: Thank you for your very encouraging comments on the paper. We agree that including text on the health care system in India will provide important context that will enable readers to better appreciate the study findings. We have therefore included a paragraph describing the rural public health system as it prevails in India (Pages 5-6 Lines 120-146). The study was carried out in rural areas of select districts in the states of Uttar Pradesh and Madhya Pradesh.

**Comment 2**. Related to the above point: an understanding of management structures of the health system could also help sharpen the recommendations. Which people in which offices should be held accountable or responsible for the findings. The recommendations (in the conclusion) need to be improved to mention which offices can be held accountable (or called upon to act)-it certainly can not be the entire government.

Response: We have in the above text described the three tier set up of the rural public health system in India. The Health Sub Centre (HSC) is the most basic as well as the most peripheral unit which is attached to a Primary Health Centre (PHC) and is the link between the community and the PHC. The PHCs serve a population of about 30,000, are staffed with one medical officer and about 14 paramedical and administrative staff and acts as a referral unit for 6 HSCs. The PHC in turn reports to the Community Health Centre (CHC) which are larger 30 bed hospitals equipped with operation theatres, labour room, x-ray and lab facilities and medical specialists. .Over and above all this are the district hospitals which serve as the tertiary referral hospital (Pages 5-6; Lines 120-146)

**Comment 3.** In the limitation, the authors mention that they needed a larger sample size. I think they are referring to more heterogeneity. The two are related but they could reconsider revising the term.

Response: We actually meant including more medical officers working in other CHCs in urban settings and PHCs, the first level health services so as to obtain a diversity of perceptions. However, we were unable to do this on account of the logistics of travel and the limited time we had within which to complete our data collection.

**Comment 4.** Under perceptions of CHWs (Page 10, line 234), they use the term "respiratory distress". This can be misleading, I suspect they want to mean a cough because that is what is managed with Cotrimoxizole. Kindly double check. If the term was misused, the maybe put it in inverted commas.

Response: We thank you for pointing this out to us. The CHWs do in fact refer to cough and to difficulties in breathing and the use of the term “respiratory distress” would perhaps be inaccurate. We have now changed this to “respiratory problems” (Page 11, Line 268)

**Comment 5**. The authors may consider creating a table that summarizes the findings. As it is now, it may be difficult to navigate quickly.

Response: We have now made revisions to the results section, reorganized the themes and have incorporated the quotes within the text. This, we believe will make for easier reading and contextualizing.

**Reviewer #2**: This is a qualitative study regarding health care providers’ perceptions regarding management of childhood pneumonia in India. The data presented is a part of a larger mixed-methods study. The paper is well-organized and clearly written. The authors provide a good description of the study methodology and within the results section, data are presented within thematic categories which emerged through data analysis. The authors also discuss their data in relation to previous studies. There are some general take-away messages, but I think it might be more useful in the discussion section to link outcomes from the study (e.g., government hospitals access to supplies, training CHWs or other providers, community education and community trust in health care providers) to specific, feasible and potentially sustainable interventions that have been successful in similar communities. Some of this is in the discussion section (e.g., allowing CHWs to dispense antibiotics for pneumonia cases – though this approach could potentially lead to more unnecessary and inappropriate use of antibiotics in communities where they are already often misused). I would also be interested in seeing some information about what questions emerged or went unanswered during the study which might be important for follow-up research in the study sites, India, and/or region.

Response: Thank you so much for your very encouraging comments. We have reorganized both the results and discussion sections and have incorporated some of your suggestions. Regarding the issue of questions that need further exploration, there is perhaps the role of private care providers in terms of what challenges they face in the delivery of pneumonia care in these rural communities and how they cope with it. Also, is there any sort of public-private partnership whereby they too serve as important links in the chain of care through early diagnosis (being immediately available), provision of basic treatment and appropriate guidance on referrals? Then there are the UCPs who by virtue of their easy access and low cost are seen as care providers at least in UP. How do they perceive their roles, can they be trained and what are the pros and cons of training this human resource could be issues that merit exploration

,

**Reviewer #3:** Thank you for the paper.

**Comment 1**. Title: Add ‘the’ after exploring

Response. This has been included

**Comment 2** Equal contribution on authorship should be reserved for first and last author otherwise it seems overused

Response: We have now revised the author contribution

Rani Mohanraj: Conceptualization, Formal analysis, Investigation, Methodology, Project administration, Supervision, Writing-Original draft

Shuba Kumar: Conceptualization, Formal analysis, Investigation, Methodology, Project administration, Supervision, Writing-review & editing

Monica Agarwal: Investigation, Project administration, Supervision

Bhavna Dinghra: Investigation, Project administration, Supervision

Saradha Suresh: Conceptualization, Investigation, Methodology, Project administration, Supervision, Writing-review & editing

**Comment 3.** State the total number of participants in FGDs, again state the number per FGD and state how the composition.

Response: We apologise for not clarifying this. We have now provided details of the number of FGDs and the number of participants in each FGD under the Results section. A total of eight FGDs with CHWs were carried out. Wherever possible we conducted separate FGDs with ASHAs and ANMs. Where their numbers were less, we combined ANMs and ASHAs in one FGD. Each FGD had an average of about 8-12 participants (total 82 participants). In UP, we carried out 5 FGDs (3 with ASHAs; n=32 and 2 with ANMs: n=14) and in MP we conducted 3 FGDs (mixed group of ASHA and ANM ; n=36) (Pages 8; Lines 206-209 )

**Comment 4:** Structure the Abstract appropriately

Response: The abstract has been prepared according to the requirements of PLOS-Global health. It must not exceed 300 words and must not include the headings, background, methodology/principal findings, and conclusions/significance. We have accordingly prepared the abstract which adds up to 296 words (Page 2; Lines-28- 50)

**Comment 5:** Seemingly some themes were deduced and not realised - that distinction should be clear e.g theme on challenges Line 67-81- I find it confusing, it seems to be the previous study that led to the current one, if that is the case then it should be better summarised and show the linkage clearly

Response: We apologize for the confusion. We have now revised and reorganized the themes and have used the Andersen and Newman model for health service utilization (2005) to explain our findings. We believe the results section is now easier to read and understand. We have included a paragraph at the end of the introduction section explaining the fact that this paper that specifically looks at HCP perspectives was a component of a larger study which included a household survey carried out with mothers of children less than 5 years of age in selected districts in the states of Uttar Pradesh, Madhya Pradesh and Tamil Nadu. The study aimed to understand care seeking behaviour for childhood pneumonia. Qualitative interviews were also carried out with a sub-sample of mothers. We have already published two papers presenting the findings from the household survey and the qualitative interviews with the mothers (Ref: **1.** Mohanraj R, Kumar S, Jayakumar S, et al. Where do mothers take their children for pneumonia care? Findings from three Indian states. PLoS One. 2019;14:e0214331; **2.** Kumar S, Mohanraj R, Dhingra B, Agarwal M, Suresh S. Optimizing Care-Seeking for Childhood Pneumonia: A Public Health Perspective. Indian Pediatr. 2021 Nov 15;58(11):1030-1035. PMID: 34837362.).For the current study we only included the HCP related data from the two states of UP and MP as both these states have a high prevalence of pneumonia and their IMR indices are also high.

**Comment 6** Methods- State the specific qualitative approach that was followed
Justify selection of the sites:

Response: We used a hybrid deductive-inductive thematic analysis strategy. To begin with we deductively developed a code book based on the Health Systems and Evaluated Need component of the Andersen and Newman framework. Following data collection, we followed the seven steps of thematic analysis as detailed by Braun and Clarke (2013) which allowed us to inductively code thereby enabling us to expand on the existing code book (Pages 7-8; Lines 187-202).

In the introduction we have explained the reasons for selection of UP and MP which was mainly because of the high infant mortality rates (IMR). The state of Tamil Nadu (TN) with lower IMR and low prevalence of childhood pneumonia served as a comparator state. For this paper, however, we have not included data from the HCPs from TN site (Pages 3; Lines 76-86)

**Comment 7** Was there any theory that guided the study? If not, what informed the development of tools? If you had no theory at point of design, acknowledge it as a limitation, however, use a theory to interpret your findings- you will achieve a better presentation and discussion of the results

Response: The Anderson and Newman’s framework of health care utilization (2005) was used to guide our study and findings. More specifically the health systems and the evaluated need component of the model were used in explaining the perspectives of the HCPs (Pages 4-5; Lines 105-117)

**Comment 8.** Upload the guides that were used

Response: Guides have been uploaded

**Comment 9.** Upload the Code book

Response: Code book has been uploaded

**Comment 10.** How was coding done? deductive and inductive?

Response: As described under comment 5 we used a hybrid deductive-inductive thematic analysis strategy. To begin with we deductively developed a code book based on the Health Systems and Evaluated Need component of the Andersen and Newman model. Following data collection, we followed the seven steps of thematic analysis as detailed by Braun and Clarke (2013) which allowed us to inductively code thereby enabling us to expand on the existing code book. This has been described under the section ‘Analysis’ (Pages 7- 8; Lines 187-202)

**Comment 11.** Results- Table 1- Group common variables like occupation- MDs, MOs. The current presentation of the table makes it difficult to follow as there are many repetitions that can be avoided if done better. For age- present the median with IQR if possible. Use either Barriers or Challenges but not both. The results need to be revised thoroughly, they are difficult to follow in the current state. You will need to have categories/subthemes under each theme and then show the quotes for each category presented. Analyse the system level results using the WHO building block framework. It will give the results better presentation and order All the results would benefit from a better presentation and analysis
Employ constant comparison across the different groups so that one is able to appreciate areas of convergence and divergence- that will highlight the results better
there is an overlap between exclusive breastfeeding and nutrition - separating them makes them difficult to be viewed as such. revisit these aspects

Response: Thank you for all your valuable suggestions. We have now deleted the demographic table which we realise was cumbersome and have conveyed the information in text form (Pages 8-9; lines 210-227). The results section has also been modified and reorganized. We also reviewed the WHO framework as suggested, which describes health systems in terms of six building blocks: (i) service delivery; (ii) health workforce; (iii) health information systems; (iv) access to essential medicines; (v) financing; and (vi) leadership/governance. However, as mentioned earlier, the primary focus of our main study was to explore care seeking behaviour for childhood pneumonia in the community. In the interest of triangulation and for obtaining a holistic picture we also included HCPs to understand their perceptions on barriers they faced in care delivery for management of childhood pneumonia. We therefore may not have the information necessary to fulfil the requirements of the WHO framework.

**Comment 12**. Discussion First paragraph should summarise the key findings in a paragraph and then discuss them from that summary. I have pended review of the discussion till after the results are revised and presented in the suggested manner.

Response: Thank you for this suggestion. We have now revised this section and have included the key findings first and have introduced additional text comparing with other studies

:
